# The ReWalk ReStore™ soft robotic exosuit: a multi-site clinical trial of the safety, reliability, and feasibility of exosuit-augmented post-stroke gait rehabilitation

**DOI:** 10.1186/s12984-020-00702-5

**Published:** 2020-06-18

**Authors:** Louis N. Awad, Alberto Esquenazi, Gerard E. Francisco, Karen J. Nolan, Arun Jayaraman

**Affiliations:** 1 Department of Physical Therapy & Athletic Training, Boston University, Boston, MA USA; 2 Wyss Institute for Biologically Inspired Engineering, Harvard University, Boston, MA USA; 3 Department of PM&R, Harvard Medical School, Spaulding Rehabilitation Hospital, Boston, MA USA; 4 Department of PM&R, MossRehab and Einstein Healthcare Network, Elkins Park, PA USA; 5 Department of PM&R, University of Texas McGovern Medical School, TIRR Memorial Hermann, Houston, TX USA; 6 Center for Mobility and Rehabilitation Engineering, Kessler Foundation, West Orange, NJ USA; 7 Department of PM&R, Rutgers New Jersey Medical School, Kessler Rehabilitation, Newark, NJ USA; 8 Department of PM&R, Northwestern University, Chicago, IL USA; 9 Shirley Ryan AbilityLab, Chicago, IL USA

**Keywords:** Stroke, Exoskeleton, Exosuit, Rehabilitation, Physical therapy, Walking

## Abstract

**Background:**

Atypical walking in the months and years after stroke constrain community reintegration and reduce mobility, health, and quality of life. The ReWalk ReStore™ is a soft robotic exosuit designed to assist the propulsion and ground clearance subtasks of post-stroke walking by actively assisting paretic ankle plantarflexion and dorsiflexion. Previous proof-of-concept evaluations of the technology demonstrated improved gait mechanics and energetics and faster and farther walking in users with post-stroke hemiparesis. We sought to determine the safety, reliability, and feasibility of using the ReStore™ during post-stroke rehabilitation.

**Methods:**

A multi-site clinical trial (NCT03499210) was conducted in preparation for an application to the United States Food and Drug Administration (FDA). The study included 44 users with post-stroke hemiparesis who completed up to 5 days of training with the ReStore™ on the treadmill and over ground. In addition to primary and secondary endpoints of safety and device reliability across all training activities, an exploratory evaluation of the effect of multiple exposures to using the device on users’ maximum walking speeds with and without the device was conducted prior to and following the five training visits.

**Results:**

All 44 study participants completed safety and reliability evaluations. Thirty-six study participants completed all five training days. No device-related falls or serious adverse events were reported. A low rate of device malfunctions was reported by clinician-operators. Regardless of their reliance on ancillary assistive devices, after only 5 days of walking practice with the device, study participants increased both their device-assisted (Δ: 0.10 ± 0.03 m/s) and unassisted (Δ: 0.07 ± 0.03 m/s) maximum walking speeds (P’s < 0.05).

**Conclusions:**

When used under the direction of a licensed physical therapist, the ReStore™ soft exosuit is safe and reliable for use during post-stroke gait rehabilitation to provide targeted assistance of both paretic ankle plantarflexion and dorsiflexion during treadmill and overground walking.

**Trial registration:**

NCT03499210. Prospectively registered on March 28, 2018.

## Introduction

Bipedal locomotion is characterized by alternating periods of single and double limb support, with ground clearance by the swing limb and propulsion by the trailing stance limb serving as crucial walking subtasks [1, 2]. Healthy individuals are able to generate an ankle dorsiflexion moment during each limb’s swing phase to lift the foot and facilitate ground clearance. They are also able to generate an ankle plantarflexion moment during each limb’s late stance phase to produce the propulsive force required to advance the limb and body [3]. In contrast, post-stroke hemiparesis results in impaired paretic dorsiflexion and plantarflexion that, in turn, hinders ground clearance and propulsion [4–8] and, ultimately, necessitates compensatory walking strategies [9, 10] that make walking more effortful and unstable [11–14].

The ReWalk ReStore™ is a soft robotic exosuit designed to augment the paretic ankle’s ability to produce both dorsiflexor and plantarflexor moments during walking. In early proof-of-concept studies conducted with a research version of the device [15, 16], exosuits were shown to facilitate immediate increases in swing phase paretic ankle dorsiflexion by an average 5 degrees [17], the propulsion force generated by the paretic limb by an average 10% [17], and the positive center of mass (COM) power generated by the paretic limb during late stance phase by an average 22% [18]. Together, these improvements in paretic limb function resulted in reduced propulsion asymmetry by 20% [17] and the asymmetry in positive COM power generated during late stance phase by 39% [18]. Also observed were immediate reductions in hip hiking and circumduction compensations of over 20% [9], reductions in the energy cost of walking by an average 10% [17, 18], faster overground walking speeds by a median 0.14 m/s, and farther walking distances during the 6-min walk test by a median 32m [19].

Building on this foundational biomechanical, physiological, and clinical research, the objective of this multi-site clinical trial was to evaluate safety, feasibility, and reliability of using exosuits during post-stroke rehabilitation in preparation for a commercial clinical application to the United States Food and Drug Administration (FDA). In contrast to previous laboratory-based research that studied the immediate effects of exosuit prototypes on clinical, biomechanical, and physiological outcomes, this translational research sought to determine the safety of clinicians and patients with post-stroke hemiparesis using the commercially-adapted ReStore™ in clinical settings, the feasibility of clinician operators applying the ReStore™ during both treadmill and over ground gait training activities, and the reliability of the technology across multiple training visits. In addition to outcomes of safety, feasibility, and device reliability, an exploratory evaluation of the impact that multiple training visits with the device have on users’ maximum walking speeds, both with and without the device, was also included.

## Methods

The ReStore™ is indicated for use by individuals with post-stroke hemiparesis undergoing stroke rehabilitation under the supervision of a licensed physical therapist. To assess the safety, device reliability, and clinical feasibility of using the ReStore™ during post-stroke gait rehabilitation, a multi-site trial was conducted. The trial included five clinical sites and 44 users with post-stroke hemiparesis. The study was approved by the Institutional Review Boards of Boston University, Spaulding Rehabilitation Hospital, The Shirley Ryan AbilityLab, TIRR Memorial Hermann Hospital, Kessler Rehabilitation Hospital, and Moss Rehabilitation Hospital. Written informed consent was secured for all participants.

### Study inclusion and exclusion criteria

Study participant eligibility requirements consisted of: (i) one-sided ischemic or hemorrhagic stroke, (ii) > 2 weeks post-stroke, (iii) age > 18 years, (iv) height between 4′8″ and 6′7″, (v) weight < 264lbs, (vi) medical clearance, (vii) ability to ambulate at least 5 ft without an AFO and with no more than minimal contact assistance, (viii) ability to follow a 3-step command, (ix) ability to fit suit components, (x) no greater than 5 degrees of ankle plantar flexion contracture, and (xi) Modified Ashworth Scale for tone at 3 or less for ankle dorsiflexor and plantarflexor muscles. Exclusion criteria included: (i) severe aphasia limiting ability to express needs or discomfort verbally or non-verbally, (ii) serious co-morbidities that interfere with ability to participate, (iii) significant Peripheral Artery Disease, (iv) colostomy bag, (v) current pregnancy, (vi) uncontrolled hypertension, (vii) participation in any other clinical trial, (viii) open wounds or broken skin at device locations requiring medical management, (ix) urethane allergies, (x) and current DVT.

### Study overview

After screening and enrollment, study participants completed up to two walking evaluations and five device exposure visits. Each exposure visit consisted of up to 20 min of overground walking practice and 20 min of treadmill walking practice while receiving assistance from the device. The visit schedule consisted of a minimum of two visits per week, with the expectation of no more than 4 weeks between the pretraining and posttraining evaluations. Actual activities and durations were dependent on each study participant’s abilities as determined by the treating physical therapist as per their usual practices. The target level for plantarflexion assistance during all active walking with the ReStore™ was 25% of the user’s bodyweight [17, 19]. The target level for dorsiflexion assistance was the minimum needed for adequate ground clearance and heel strike, as determined visually by the physical therapist.

### Device overview

**Fig. 1:**
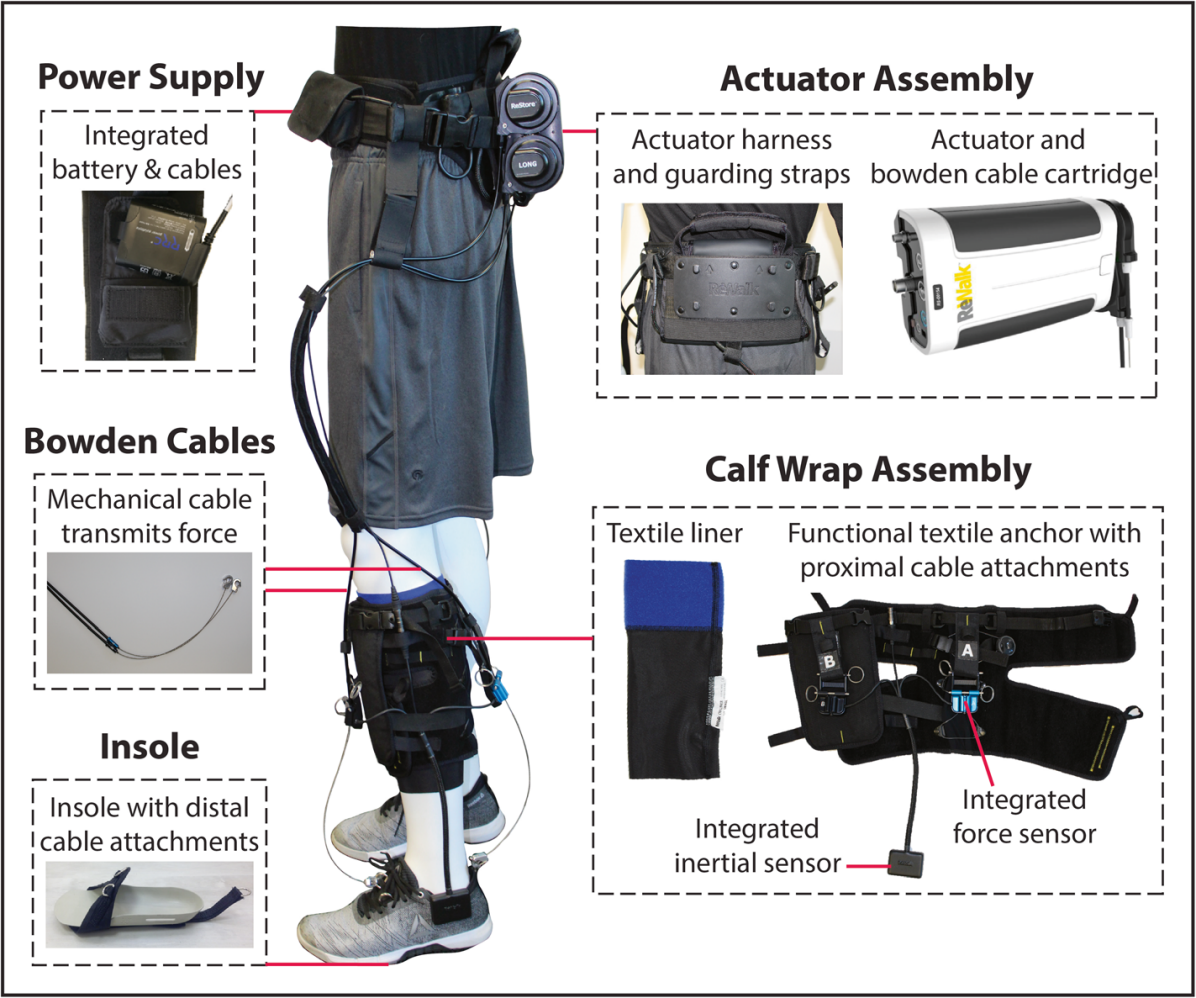
The ReWalk ReStore™ consists of an actuator assembly, calf wrap assembly, and shoe insole. A power supply is integrated into the actuator assembly. Bowden cables span these components to transmit assistive forces generated by the actuator to the ankle. More specifically, two Bowden cables are used in the ReStore™, each having attachment points proximal (on the Calf Wrap Assembly) and distal (on the Insole) to the ankle. One of the cables is located anterior to the ankle and the other is located posterior to the ankle. When the anterior cable is retracted, an ankle dorsiflexion torque is produced. When the posterior cable is retracted, an ankle plantarflexion torque is produced

**Fig. 2:**
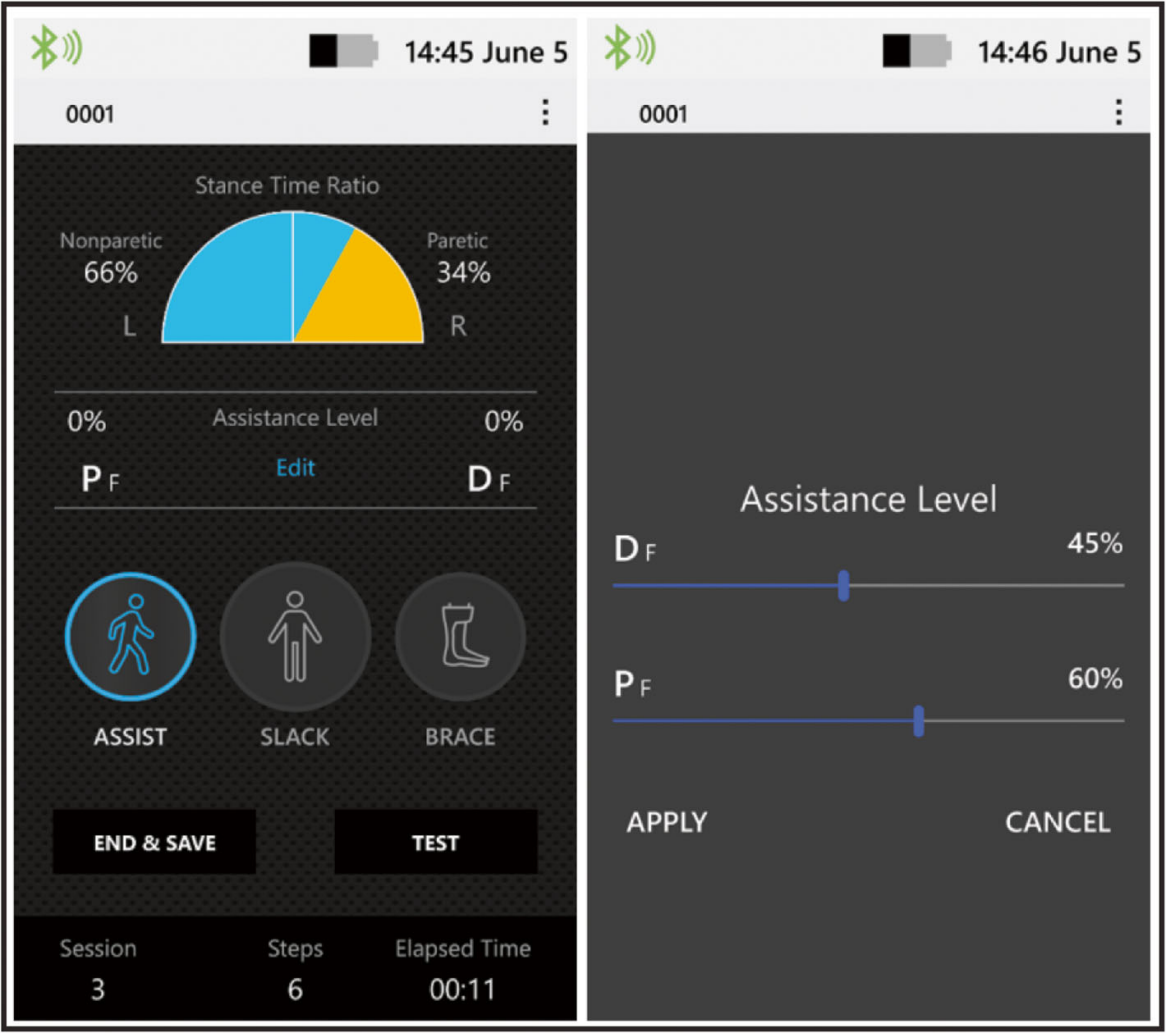
*Left -* The ReWalk ReStore™ graphical user interface allows three modes of use: Assist, where ankle plantarflexion and dorsiflexion are actively assisted; Slack, where the device is made transparent to the user; Brace, where the ankle dorsiflexion cable is tensioned throughout the entire gait cycle to mimic an ankle foot orthosis during swing phase without hindering ankle dorsiflexion during the stance phase. In addition, the user’s stance time symmetry is shown and updated on a step-by-step basis. *Right -* The amplitude of ankle plantarflexion and dorsiflexion assistance can be modified in real-time using a visual slide ruler with a 0 to 100% scale. 100% plantarflexion assistance corresponds to a force equal to 25% of the wearer’s bodyweight. 100% dorsiflexion assistance corresponds to the maximum allowed cable travel distance (50 mm). For the study, the target level for plantarflexion assistance was 100% (i.e., 25% of the wearer’s bodyweight) and the target level for dorsiflexion assistance was the minimum needed for adequate ground clearance and heel strike, as determined visually by the physical therapist. See previous work [19] for visual depiction of the forces applied by the exosuit

The exosuit consists of motors worn at the waist that generate mechanical forces that are transmitted by cables to attachment points located proximally on a functional textile worn around the calf and distally on a shoe insole (Fig. 1). The overall weight of the exosuit is approximately 5kgs, with the vast majority of the weight located proximally in the actuation pack worn at the waist. Each functional textile contains a detachable liner that can be washed. For users who require medio-lateral ankle support in addition to ankle plantarflexion and dorsiflexion assistance, an optional textile component that prevents ankle inversion without restricting dorsiflexion and plantarflexion can also be used. Inertial sensors that attach to a patient’s shoes measure gait events and automate the independent timing of the active ankle plantarflexion and dorsiflexion assistance provided by the ReStore™ as previously described [16]. Load cell sensors located at the end of each cable are used to monitor the interaction between user and exosuit and ensure that the target level of assistance is achieved [16, 17]. A hand-held device with a graphical interface allows clinicians to monitor patients’ performance and select and progress, in real-time, the assistance parameters (Fig. 2).

### Outcomes and analyses

The primary endpoint of safety was assessed as the frequency of device-related adverse events during the study, including device-related serious adverse events (as determined by the clinical investigators) and falls. Secondary endpoints of clinician safety and device reliability were assessed as the frequency of device-related injuries experienced by physical therapists during the study and device malfunctions during device usage, respectively. Clinical feasibility was assessed, in part, using two custom questionnaires, with each question scored on a scale from 1 to 5. One questionnaire was provided to the 36 study participants who completed all planned visits and activities (see Additional file 1), with a score of 5 indicating they were “very satisfied” and a score of 1 indicating they were “not satisfied at all”. The other questionnaire was provided to the 14 licensed physical therapists who operated the ReStore™ across study sites (see Additional file 2), with a score of 5 indicating “Strongly Agree” and a score of 1 indicating “Strongly Disagree”.

In addition to the primary and secondary endpoints of safety, device reliability, and clinical feasibility, an exploratory assessment of changes in unassisted (i.e., no exosuit) and exosuit-assisted maximum walking speed, measured using the 10-m walk test before and after the five exposure visits, was conducted. It should be noted that this study’s exploratory assessment of changes in speed was included to evaluate the therapeutic potential of using the ReStore™ as a rehabilitation robot in advance of future clinical efficacy trials. This study was not designed to assess immediate device efficacy (i.e., versus a no device control) as in previous studies of the exosuit technology that included individualized tuning of the assistance parameters and dedicated device exposure visits [9, 17, 19].

Study participants were allowed to use their assistive device (e.g., a cane) and Ankle Foot Orthosis (AFO) during unassisted walking speed evaluations if using these devices were required for safety. Although AFOs were not usable during exosuit-assisted walking evaluations (due to incompatibility with the active assistance of ankle plantarflexion provided by the ReStore™), if a cane was used during unassisted testing, a cane was similarly used during exosuit-assisted testing. Study participants’ walking speeds before and after the multi-visit exposure to walking with the ReStore™ were compared to clinically meaningful difference scores [20–22] and using pairwise comparisons. Study participants were also dichotomized into those who required the use of an AFO or assistive device during evaluations and those who did not require the use of an AFO or assistive device. Differences in walking speed improvements between these groups were compared using independent t-tests. Alpha was set to 0.05 for all analyses.

## Results

Forty-four study participants were enrolled in the study (see Table 1 for baseline characteristics and device component sizes used across participants with different body types). Of these individuals, 60% had an ischemic stroke and 41% were right hemiparetic. Their average age was 54.8 years, they were 7.3 years post-stroke, and they walked with an average comfortable walking speed of 0.82 m/s. The vast majority (73%) customarily used passive assistive technology (i.e., AFOs, canes, walkers) for community mobility. All 44 study participants completed testing activities with the device and were thus included in the evaluation of the safety endpoints. Eight individuals withdrew early from the study. These individuals were not observed to be different in any baseline characteristic from the individuals who completed all study activities (*p’s* > 0.05). Thirty-six device users completed all planned visits and activities and were included in the analysis of the secondary and exploratory clinical endpoints. Of the eight individuals who withdrew early from the study, four requested the withdrawal and one was removed after being admitted for an emergency surgery that was not related to the device or study. The remaining three individuals were withdrawn at the request of the device manufacturer due to the need to update the device software. Two of these individuals were ultimately re-enrolled.

**Table 1:** Participant characteristics and ReStore Exosuit Sizing

Study Subject	Stroke Type	Side of Paresis	Sex	Age (y)	Chronicity (y)	Height (in)	Weight (lbs)	Walking Speed (m/s)^**b**^	Assistive Device	Calf Wrap Size	Liner Size	Insole Size	Lateral Support
1^a^	Ischemic	Left	Female	48	11.9	74.0	216	0.92	AFO & Cane	Small	Small	Large	Yes
2^a^	Hemorrhagic	Right	Female	36	1.8	71.0	164	1.21	Ankle Brace	Medium	Medium	Medium	Yes
3^a^	Unknown	Right	Male	50	6.2	72.0	180	1.38	None	Medium	Medium	Medium	Yes
4^a^	Ischemic	Right	Female	65	32.7	67.0	128	1.14	AFO & Cane	Small	Small	Medium	No
5^a^	Ischemic	Right	Female	47	13.4	68.5	113	0.95	AFO & Cane	Small	Small	Medium	No
6^a^	Hemorrhagic	Left	Male	62	4.9	65.0	165	1.09	Cane	Small	Small	Medium	No
7^a^	Ischemic	Left	Male	67	0.6	66.0	174	0.77	Rollator	Medium	Medium	Medium	No
8^a^	Hemorrhagic	Left	Female	49	12.7	65.0	155	0.91	None	Medium	Medium	Small	No
9^a^	Ischemic	Left	Female	45	6.9	64.0	201	0.72	AFO	Small	Small	Small	No
10^a^	Hemorrhagic	Left	Male	53	20.6	65.0	214	0.96	AFO	Medium	Medium	Large	No
11^a^	Hemorrhagic	Left	Female	42	11.4	63.5	177	0.99	None	Large	Large	Medium	No
12^a^	Ischemic	Left	Female	55	1.3	64.0	180	0.79	AFO & Cane	Medium	Medium	Medium	No
13^a^	Ischemic	Right	Female	72	1.1	63.0	140	1.08	None	Medium	Medium	Small	No
14	Ischemic	Left	Male	47	5.8	74.0	246	0.87	AFO	Large	Large	Large	No
15^a^	Ischemic	Left	Female	70	2.2	64.0	160	0.29	AFO	Small	Small	Medium	No
16^a^	Ischemic	Left	Male	61	13.8	71.0	188	0.35	WalkAide & Cane	Medium	Small	Large	Yes
17^a^	Hemorrhagic	Right	Female	46	18.4	65.0	220	0.54	WalkAide & Cane	Large	Large	Medium	No
18^a^	Ischemic	Right	Male	62	1.1	72.0	225	1.14	AFO	Large	Large	Large	No
19^a^	Ischemic	Left	Female	57	1.8	64.0	142	0.11	AFO & Cane	Small	Small	Medium	Yes
20^a^	Hemorrhagic	Left	Male	50	6.8	69.2	141	0.68	None	Medium	Small	Large	No
21^a^	Hemorrhagic	Left	Male	55	3.2	71.0	163	0.50	None	Small	Small	Large	No
22^a^	Ischemic	Right	Female	27	2.1	64.0	157	0.90	None	Large	Large	Small	No
23^a^	Ischemic	Left	Male	68	0.9	73.0	194	1.00	AFO & Cane	Small	Medium	Large	No
24^a^	Hemorrhagic	Right	Male	69	4.7	69.5	169	0.56	AFO	Large	Medium	Large	No
25^a^	Ischemic	Left	Female	52	11.8	65.0	135	0.91	AFO	Small	Small	Small	No
26^a^	Ischemic	Right	Male	66	2.3	69.0	190	0.99	AFO & Cane	Large	Medium	Large	No
27	Hemorrhagic	Left	Male	59	6.7	69.5	163	1.00	None	Medium	Medium	Large	No
28	Ischemic	Left	Male	66	9.0	66.7	249	0.74	AFO & Cane	Extra Large	Large	Large	No
29^a^	Ischemic	Right	Female	50	5.3	64.8	181	0.51	AFO & Cane	Large	Medium	Medium	No
30	Hemorrhagic	Right	Female	33	6.9	65.5	152	1.12	None	Medium	Medium	Medium	No
31	Ischemic	Left	Male	55	14.5	67.3	162	0.72	AFO	Small	Small	Medium	Yes
32	Ischemic	Right	Male	64	16.2	72.0	236	0.98	AFO	Medium	Medium	Large	No
33^a^	Hemorrhagic	Right	Male	36	7.5	68.3	250	0.88	AFO	Large	Large	Medium	No
34^a^	Ischemic	Left	Male	66	9.4	68.0	233	0.73	AFO & Cane	Large	Large	Large	No
35^a^	Hemorrhagic	Left	Male	60	7.0	70.6	163	1.10	Cane	Medium	Small	Large	No
36	Ischemic	Right	Male	67	3.4	67.7	175	0.38	Cane & Rollator	Medium	Medium	Medium	No
37^a^	Ischemic	Left	Male	46	12.9	70.1	179	0.85	None	Medium	Medium	Medium	Yes
38^a^	Hemorrhagic	Left	Male	50	1.5	74.0	211	0.63	Cane	Medium	Medium	Large	No
39	Ischemic	Left	Male	53	0.4	70.0	196	0.58	Cane	Medium	Medium	Medium	No
40^a^	Ischemic	Right	Male	36	0.6	71.0	231	0.95	None	Large	Large	Medium	No
41^a^	Ischemic	Left	Male	60	1.7	66.0	179	0.96	None	Medium	Medium	Small	No
42^a^	Hemorrhagic	Left	Male	68	6.0	67.0	178	0.50	Cane	Medium	Medium	Large	No
43^a^	Ischemic	Right	Female	56	0.9	63.0	163	0.93	AFO	Small	Small	Small	Yes
44^a^	Ischemic	Right	Male	66	9.8	72.0	199	0.89	AFO	Medium	Medium	Medium	No

^a^*N* = 36 subset of study participants who completed all planned study visits and were included in the analysis of secondary and exploratory endpoints

^b^Usual walking speed assessed at baseline

### Device usage

On average, device users were exposed to 311.4 ± 114.4 total minutes of walking with the ReStore™. Plantarflexion and dorsiflexion assistance levels were set by clinician operators to, on average, 92.0 ± 15.4% and 63.1 ± 21.7%, respectively. These assistance levels varied minimally across days, ranging from a minimum of 91.3 ± 15.1% plantarflexion assistance on training day 1 to a maximum of 94.3 ± 14.9% plantarflexion assistance on training day 5. Similarly, dorsiflexion assistance ranged from a minimum of 64.0 ± 20.4% on training day 5 to a maximum of 65.3 ± 23.1% on training day 2.

### Clinical feasibility

Average satisfaction ratings for the 36 study participants who completed all planned visits and activities were between “quite satisfied” (i.e., 4) and “very satisfied” (i.e., 5) (see Supplementary Table 1). Study participants indicated that the categories of Effectiveness, Comfort, Ease of Use, and Safety were most important to them. Study participants respectively gave these categories the following average ratings: 4.3 ± 1.1, 3.9 ± 1.1, 4.0 ± 1.1, and 4.3 ± 1.1.

Average satisfaction ratings for the 14 licensed physical therapists who operated the ReStore™ were between “neither agree nor disagree” (i.e., 3) and “strongly agree” (i.e., 5) (see Supplementary Table 2). Questions related to ease of device operation and the ability to provide appropriate supervision and guarding of the subject while using the device received the highest physical therapist ratings of 4.3 ± 0.83 and 4.3 ± 0.91, respectively. The lowest average rating provided by the study physical therapists was 3.1 ± 0.95 and was in regards to the time spent donning/doffing the device.

### Safety data

Device-related adverse events occurred in less than 10.0% of study visits. The majority of adverse events were considered mild in severity (i.e., did not require intervention or treatment and resolved uneventfully). There were no device-related falls or serious adverse events in the study. The most frequent device-related adverse events were pain in the lower extremity (11 events) and skin abrasions (7 events). Other adverse events reported include contusion (2 events), erythema (2 events), blister (1 event), arthralgia (1 event), neuralgia (1 event), limb discomfort (1 event), and joint swelling (1 event). It should be noted that approximately 70% into the trial, it was suspected by the device manufacturer that the majority of adverse events were related to improper fitting of specific device components. The device manufacturer thus conducted a mandatory re-training with updated training materials at all study sites, after which the rate of adverse events dropped from 13.5% of the first 193 study visits to 1.3% of the final 75 study visits.

To assess the safety of the ReStore™ for use by clinicians, device-related injuries to the clinicians administering the gait training were tracked throughout the duration of the study. There were no reports of any injuries to the clinicians; however, there was one reported instance of an assistant sustaining a bruise following dropping the device on her thigh while attempting to set up the device. Medical intervention was not necessary, and the bruise resolved on its own.

### Device reliability data

Device malfunctions were reported to have occurred in 11.6% of study visits. A total of six device malfunctions were encountered during the first two visits of the study, three of which were related to the actuation unit and three of which were related to the handheld device. None of these device malfunctions resulted in adverse events. In response, the device manufacturer paused the study to implement a device software update. Following the device update, the most common types of malfunction were related to sensor connectivity (3.4% of visits) and usability issues with the functional textile anchor (3.4% of visits) and the underlying liner (3.0% of visits). Several malfunctions likely linked to software robustness were also reported during 3.4% of visits. These malfunctions included issues related to device error messages and alerts and Bluetooth connectivity. Additionally, for one study participant, it was reported that the device’s dorsiflexion assistance appeared to behave abnormally. These device malfunctions did not result in any adverse events and the majority of malfunctions were resolved by restarting the device or readjusting device components. Only three device malfunctions resulted in substantial time spent troubleshooting the issue and the need to alter the originally planned gait training activities. Four device malfunctions ultimately required components to be repaired or replaced by the device manufacturer.

### Exploratory clinical data

**Fig. 3:**
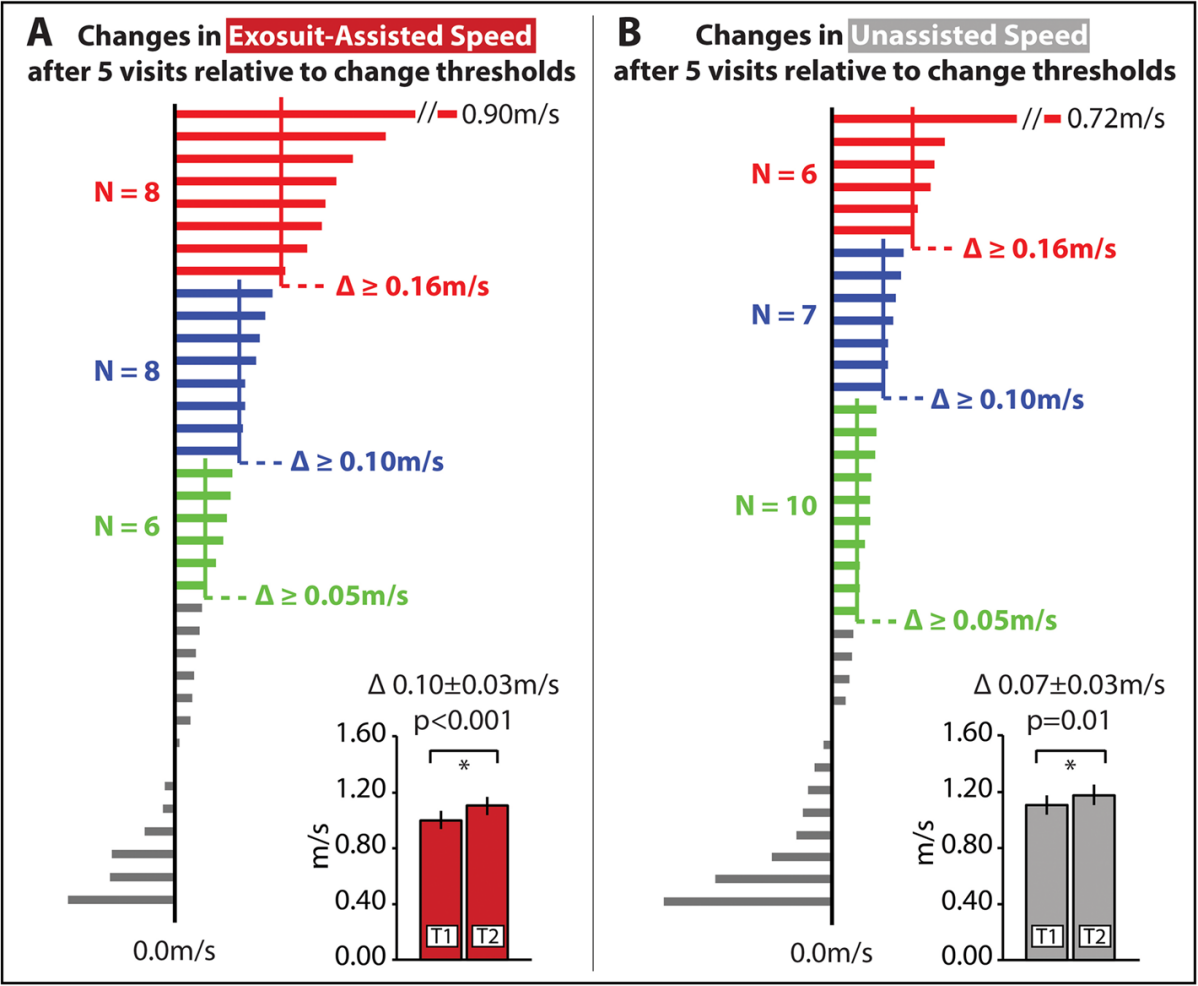
Individual subject and group-level changes in (**a**) exosuit-assisted and (**b**) unassisted maximum walking speeds after 5 days of walking practice with the ReStore™ relative to different walking speed change thresholds reported in the literature: 0.05 m/s (small meaningful change), 0.10 m/s (large meaningful change), and 0.16 m/s [20]. T1 – visit 1; T2 – visit 7 *Error bars are Standard Error*

Walking speed evaluations were performed on visits one and seven, with episodes of walking practice occurring in the five interim visits. Following the 5 days of walking practice with the ReStore™, study participants presented with an average increase in their exosuit-assisted maximum walking speed of 0.10 ± 0.03 m/s (*p* < 0.001) (Fig. 3a). Clinically meaningful difference scores previously reported for walking speed have ranged from a small meaningful change of 0.05 m/s and large meaningful change scores ranging from 0.10 m/s to 0.16 m/s [20–22]. After only 5 days of walking practice with the ReStore™, approximately 61% of study participants increased their exosuit-assisted maximum walking speed by the lower bound of 0.05 m/s, 44% increased by 0.10 m/s, and 22% surpassed the higher bound of 0.16 m/s.

Study participants also presented with an average 0.07 ± 0.03 m/s (*p* = 0.01) increase in their unassisted maximum walking speed (Fig. 3b). After only 5 days of walking practice with the ReStore™, 64% achieved the small meaningful change of 0.05 m/s, 36% increased by 0.10 m/s, and 17% surpassed the 0.16 m/s threshold.

**Fig. 4:**
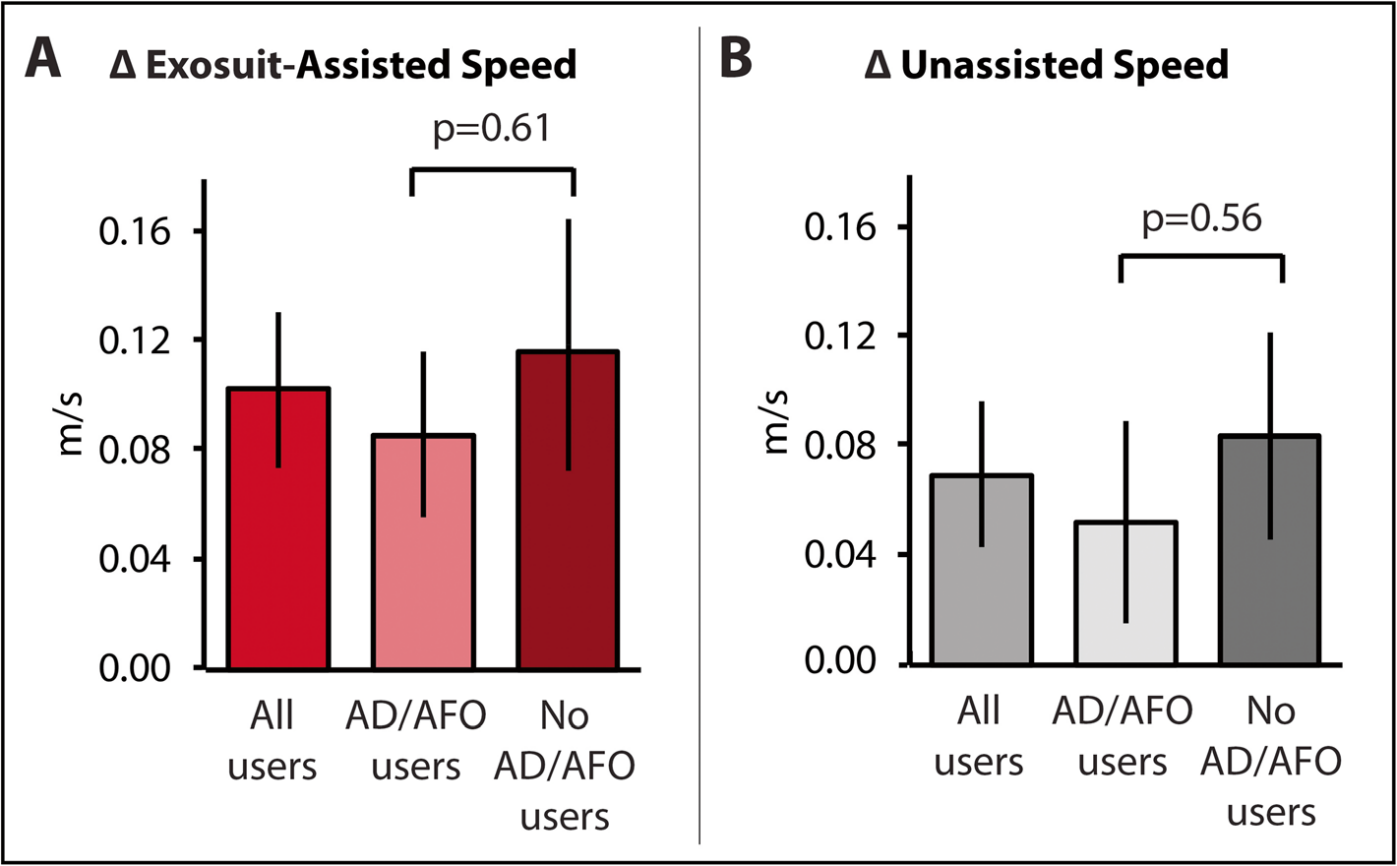
The magnitude of improvements in (**a**) exosuit-assisted and (**b**) unassisted maximum walking speed did not significantly differ between the participant subset that required the use of an AFO or cane for the walking evaluations (*n* = 16) versus the subset that did not require use of an assistive device (*n* = 20) *Error bars are Standard Error*

Sixteen of the 36 study participants required the use of an AFO or cane during the walking evaluations. We did not observe differences in either the exosuit-assisted or unassisted maximum walking speed increases across these participant subsets (Fig. 4).

## Discussion

This study builds on prior clinical, biomechanical, and physiological evaluations of soft robotic exosuits [19], demonstrating that, when used under the direction of a licensed physical therapist, the ReStore™ soft exosuit is safe and reliable for use during post-stroke gait rehabilitation. In people with post-stroke hemiparesis, the ReStore™ acts to modify a user’s walking pattern by providing ankle plantarflexion and dorsiflexion assistive forces in parallel with the underlying paretic muscles. Unlike rigid exoskeletons that are powerful enough to move the limbs without user input [23, 24], exosuit-generated forces must work in synchrony with the user’s movements to improve their walking. Given the stability deficits characteristic of the post-stroke population [14] and the potential for exosuit-generated forces to negatively perturb the user, an evaluation of the safety and reliability of clinicians operating the device during post-stroke gait rehabilitation was necessary.

Findings of no device-related falls or serious adverse events and a low rate of device malfunctions across 44 device users who completed an average of 311 min of gait rehabilitation with the device demonstrate the safety and reliability of using the ReStore™ during post-stroke rehabilitation. However, the importance of ensuring proper fit and use of the technology by supervising clinicians is highlighted by the incidental finding of a dramatic reduction in fit-related adverse events (e.g., lower extremity pain and skin abrasions) after a device re-training that was conducted at all clinical sites. The relatively high user and physical therapist satisfaction reported is promising; however, clinical centers that adopt the technology should consider site-specific training and use protocols to ensure staff are properly trained. Future generations of the soft robotic exosuit technology meant for home and community use will require a substantial development effort to enable easy donning and management of device components by patients and their caregivers.

In addition to the study’s primary and secondary objectives of safety and device reliability, we conducted an exploratory evaluation of the effect of multiple exposures to using the device on users’ maximum walking speeds with the device. We focused this analysis on users’ maximum walking speed as it is a measure of speed capacity. We found that 5 days of device exposure resulted in improvements in exosuit-assisted maximum walking speed, suggesting increased proficiency in using the Restore™ with practice. Moreover, we found that users who used an AFO or cane during the walking evaluations presented with a similar improvement magnitude as those who were able to complete testing without an AFO or cane, highlighting the robustness of these effects and the compatibility of the ReStore™ with a cane, when required.

The 5 days of walking practice with the ReStore™ also resulted in an increase in users’ unassisted maximum walking speed. With only 5 days of walking practice provided and no control group included in the study, this exploratory evaluation of changes in walking speed speaks to rehabilitative potential, not efficacy. It is noteworthy that 36% of study participants achieved a large meaningful increase (i.e., ≥ 0.10 m/s) in their unassisted maximum walking speed after only 5 days of training. Taken together with previous reports of soft robotic exosuits facilitating immediate improvements in the mechanics, energetics [9, 17, 18], walking speed, and walking distance [19] of individuals post-stroke, this multi-site safety and reliability study motivates future controlled efficacy investigations of the durability of therapeutic benefits that may arise from repeated training sessions, and thus serves to advance the exosuit technology from the lab to the clinic.

Importantly, the timing of the delivered assistive forces used in this study was constrained to the device’s default settings. This approach contrasts with prior studies of the exosuit technology that have used individualized assistance parameters based on different motion analysis techniques (i.e., measuring the energy cost of walking [17] or ground reaction forces [25]); however, these variables are not easily measured in the clinical environment and it is not clear which clinically-accessible outcomes should guide the tuning of device parameters for rehabilitative applications. Although individualization of the timing of the exosuit assistance was beyond the scope of this safety and feasibility study, given the heterogeneity of post-stroke impairment, individualizing exosuit-generated assistance to the unique needs of post-stroke users should be considered for future clinical efficacy studies of the technology and remains a crucial open question for the field.

## Conclusions

Prior work has demonstrated that soft robotic exosuits can provide targeted assistance of paretic ankle plantarflexion and dorsiflexion during hemiparetic walking to improve gait mechanics and energetics and increase walking speed and distance. This multi-site clinical trial builds on this prior work by presenting safety, reliability, and feasibility data related to the use of the technology by licensed physical therapists to support post-stroke rehabilitation. The findings of this trial advance the translation of soft robotic exosuits from the laboratory to the clinic and motivate future controlled efficacy trials.

## Supplementary information

**Additional file 1.** Study Participant Satisfaction Questionnaire.

**Additional file 2.** Physical Therapist Satisfaction Questionnaire.

**Additional file 3: Table S1.** Study Participant Questionnaire Responses. **Table S2.** Physical Therapist Questionnaire Responses.

## Data Availability

Data can be provided upon written request to the authors.
