# Supplementary material for: The ReWalk ReStore™ soft robotic exosuit: a multi-site clinical trial of the safety, reliability, and feasibility of exosuit-augmented post-stroke gait rehabilitation
Source: J Neuroeng Rehabil. 2020 Jun 18;17:80. doi: 10.1186/s12984-020-00702-5 (PMC7301475; doi:10.1186/s12984-020-00702-5)
Supplement: Supplementary file 1 — Additional file 1. Study Participant Satisfaction Questionnaire. [file 12984_2020_702_MOESM1_ESM.doc]

| Date of Questionnaire://20 dd/mmm/yyyy  [To be performed at Day 7 Visit] |
| --- |

The purpose of the **QUEST** questionnaire is to evaluate how satisfied you are with your assistive device (Restore device) and the related services you experienced. The questionnaire consists of 12 satisfaction items.

For each of the 12 items, rate your satisfaction with your assistive device and the related services you experienced by using the following scale of 1 to 5.

| **1** | **2** | **3** | **4** | **5** |
| --- | --- | --- | --- | --- |
| Not satisfied  at all | Not very satisfied | More or less satisfied | Quite satisfied | Very satisfied |

Please circle or mark the **one number** that best describes your degree of satisfaction with each of the 12 items.

Do not leave any question unanswered.

For any item that you were not "very satisfied", please comment in the section ***comments***.

Thank you for completing the QUEST questionnaire.

| **1** | **2** | **3** | **4** | **5** |
| --- | --- | --- | --- | --- |
| Not satisfied  at all | Not very satisfied | More or less satisfied | Quite satisfied | Very satisfied |

| **ASSISTIVE DEVICE (RESTORE DEVICE)**  ***How satisfied are you with*** | |
| --- | --- |
| 1. the **dimensions** (size, height, length, width) of your assistive device? *Comments:* | 1 2 3 4 5 |
| 1. the **weight** of your assistive device? *Comments:* | 1 2 3 4 5 |
| 1. the **ease in adjusting** (fixing, fastening) the parts of your assistive device? *Comments:* | 1 2 3 4 5 |
| 1. how **safe and secure** your assistive device is? *Comments*: | 1 2 3 4 5 |
| 1. the **durability** (endurance, resistance to wear) of your assistive device? *Comments:* | 1 2 3 4 5 |
| 1. how **easy** it is to use your assistive device? *Comments:* | 1 2 3 4 5 |
| 1. how **comfortable** your assistive device is? *Comments:* | 1 2 3 4 5 |
| 1. how **effective** your assistive device is (the degree to which your device meets your needs)? *Comments:* | 1 2 3 4 5 |

Below is the list of the same 8 satisfaction items. PLEASE SELECT THE THREE ITEMS that you consider to be the most important to you. Please put an X in the 3 boxes of your choice.

1. Dimensions

2. Weight

3. Adjustments

4. Safety

5. Durability

6. Easy to use

7. Comfort

8. Effectiveness

Explain why the 3 satisfaction items were chosen: __________________________
