# Supplementary material for: The ReWalk ReStore™ soft robotic exosuit: a multi-site clinical trial of the safety, reliability, and feasibility of exosuit-augmented post-stroke gait rehabilitation
Source: J Neuroeng Rehabil. 2020 Jun 18;17:80. doi: 10.1186/s12984-020-00702-5 (PMC7301475; doi:10.1186/s12984-020-00702-5)
Supplement: Supplementary file 2 — Additional file 2. Physical Therapist Satisfaction Questionnaire. [file 12984_2020_702_MOESM2_ESM.doc]

| Date of Questionnaire://20 dd/mmm/yyyy [To be performed at once all subjects are complete for this PT] |
| --- |

Total # of subjects who completed the study with this PT: ____________

Subject number(s): _______________________________

The purpose of this survey is to collect feedback from research Physical Therapists who are involved in this study. This survey should be completed after all subjects have completed the study. For each of the statements below, please indicate how strongly you agree or disagree with each statement, using the following scale:

| **1** | **2** | **3** | **4** | **5** |
| --- | --- | --- | --- | --- |
| Strongly  Disagree | Moderately Disagree | Neither Agree nor Disagree | Moderately  Agree | Strongly  Agree |

- Please circle **one number** which best describes your answer.
- Do not leave any questions unanswered.
- For any statement that you do not “Strongly Agree” with, please explain your answer in the **comments** section.

| In general, how strongly do you agree or disagree with the statements below: | Please Circle One: |
| --- | --- |
| 1. The training I received adequately prepared me to use the device with subjects. | 1 2 3 4 5 |
| 1. The amount of time spent donning/doffing the device is feasible for use in clinical practice. | 1 2 3 4 5 |
| 1. I am satisfied with the ease of operation of the device through the user interface. | 1 2 3 4 5 |
| 1. I was able to adjust the device settings to address the unique needs of individual subjects. | 1 2 3 4 5 |
| 1. Using the device did not interfere with my ability to provide appropriate supervision and guarding of the subject throughout all sessions. | 1 2 3 4 5 |
| 1. The device was compatible with gait training activities. | 1 2 3 4 5 |
| 1. I felt that the device had a positive impact on the subjects’ walking performance. | 1 2 3 4 5 |
| 1. This device would be useful in my clinical practice. | 1 2 3 4 5 |
| 1. I would recommend this device to other PTs. | 1 2 3 4 5 |

Comments: ______________________________________________
