# Supplementary material for: The ReWalk ReStore™ soft robotic exosuit: a multi-site clinical trial of the safety, reliability, and feasibility of exosuit-augmented post-stroke gait rehabilitation
Source: J Neuroeng Rehabil. 2020 Jun 18;17:80. doi: 10.1186/s12984-020-00702-5 (PMC7301475; doi:10.1186/s12984-020-00702-5)
Supplement: Supplementary file 3 — Additional file 3: Table S1. Study Participant Questionnaire Responses. Table S2. Physical Therapist Questionnaire Responses. [file 12984_2020_702_MOESM3_ESM.docx]

**Supplementary Table 1: Study Participant Questionnaire Responses**

| **Study Participant Questionnaire Response Ratings^1^** | **Average Rating**  **Mean ± SD** |
| --- | --- |
| *The dimensions (size, height, length, width) of your assistive device?* | 4.2 ± 1.2 |
|  |  |
| *The weight of your assistive device?* | 4.2 ± 1.0 |
|  |  |
| *The ease in adjusting (fixing, fastening) the parts of your assistive device?* | 4.0 ± 1.1 |
|  |  |
| *How safe and secure your assistive device is?* | 4.6 ± 0.6 |
|  |  |
| *The durability (endurance, resistance to wear) of your assistive device?* | 4.4 ± 0.8 |
|  |  |
| *How easy it is to use your assistive device?* | 4.2 ± 1.2 |
|  |  |
| *How comfortable your assistive device is?* | 3.9 ± 1.1 |
|  |  |
| *How effective your assistive device is (the degree to which your device meets your needs)?* | 4.3 ± 1.1 |
|  |  |
| **Overall Score:** | **33.8 ± 6.1** |
| **Most Important Item Selection^2^** | **N (%)** |
| Effectiveness | 25 (69.4%) |
| Comfort | 19 (52.8%) |
| Easy to use | 18 (50.0%) |
| Safety | 16 (44.4%) |
| Weight | 9 (25.0%) |
| Adjustments | 8 (22.2%) |
| Durability | 7 (19.4%) |
| Dimensions | 6 (16.7%) |

^1^ At the end of the study, the 36 study participants who completed all training visits and activities with the ReStore^TM^ were asked to rate their satisfaction with their experience with the ReStore using a 1 to 5 scale, with 1 = “not satisfied” at all and 5 = “very satisfied”.

^2^ Subjects were asked to rate the three items that they considered to be the most important.

**Supplementary Table 2: Physical Therapist Questionnaire Responses**

| **Physical Therapist Questionnaire Response Ratings^1^** | **Average Rating**  **Mean ± SD** |
| --- | --- |
| *The training I received adequately prepared me to use the device with subjects* | 3.9 ± 1.20 |
|  |  |
| *The amount of time spent donning/doffing the device is feasible for use in clinical practice* | 3.1 ± 0.95 |
|  |  |
| *I am satisfied with the ease of operation of the device through the user interface* | 4.3 ± 0.83 |
|  |  |
| *I was able to adjust the device settings to address the unique needs of individual subjects* | 3.9 ± 0.83 |
|  |  |
| *Using the device did not interfere with my ability to provide appropriate supervision and guarding of the subject throughout all sessions* | 4.3 ± 0.91 |
|  |  |
| *The device was compatible with gait training activities* | 3.8 ± 0.97 |
|  |  |
| *I felt that the device had a positive impact on the subjects’ walking performance* | 3.6 ± 0.84 |
|  |  |
| *This device would be useful in my clinical practice* | 3.4 ± 0.94 |
|  |  |
| *I would recommend this device to other PTs* | 3.4 ± 0.94 |
|  |  |

^1^ At the end of the study, the 14 licensed physical therapists who operated the ReStore^TM^ were asked to rate their satisfaction with the ReStore using a 1 to 5 scale, with 1 = “strongly disagree” and 5 = “strongly agree”.
